# Stroke sensitivity in the aged: sex chromosome complement vs. gonadal hormones

**DOI:** 10.18632/aging.100997

**Published:** 2016-07-10

**Authors:** Louise D. McCullough, Mehwish A. Mirza, Yan Xu, Kathryn Bentivegna, Eleanor B. Steffens, Rodney Ritzel, Fudong Liu

**Affiliations:** ^1^ Department of Neurology, University of Texas Health Science Center at Houston McGovern Medical School, Houston, TX 77030, USA; ^2^ Department of Neuroscience, University of Connecticut Health Center, Farmington, CT 06030, USA

**Keywords:** ischemic stroke, sex difference, chromosome, hormone, immune response, microglia

## Abstract

Stroke is a sexually dimorphic disease. Elderly women not only have higher stroke incidence than age-matched men, but also have poorer recovery and higher morbidity and mortality after stroke. In older, post-menopausal women, gonadal hormone levels are similar to that of men. This suggests that tissue damage and functional outcomes are influenced by biologic sex (XX vs. XY) rather than the hormonal milieu at older ages. We employed the Four Core Genotype (FCG) mouse model to study the contribution of sex chromosome complement and gonadal hormones to stroke sensitivity in aged mice in which the testis determining gene (Sry) is removed from the Y chromosome, allowing for the generation of XX males and XY females. XXF, XXM, XYF, XYM and XYwt aged mice were subjected to middle cerebral artery occlusion (MCAO). XXF and XXM mice had significantly larger infarct volumes than XYF and XYM cohorts respectively. There was no significant difference in hormone levels among aged FCG mice. XXF/XXM mice also had more robust microglial activation and higher serum levels of pro-inflammatory cytokines than XYF/XYM cohort respectively. We concluded that the sex chromosome complement contributes to ischemic sensitivity in aged animals and leads to sex differences in innate immune responses.

## INTRODUCTION

Clinically, ischemic stroke is recognized as a sexually dimorphic disease. Before menopause, women have a lower risk of stroke relative to men of the same age [1], uncovering a male phenotype of “ischemic-sensitivity” that has been attributed to the lack of the protective effects of estrogen (E_2_). After menopause, the incidence of stroke in women increases [2], coincident with diminished circulating levels of estrogens and progesterone. However, ischemic sexual dimorphism is not explained solely by circulating hormone levels. Stroke incidence in women does not begin to climb until decades after the natural menopause, suggesting there are hormone independent effects on ischemic sensitivity. Elderly women not only have higher stroke incidence than age-matched men, but also have poorer recovery, higher morbidity and mortality once a stroke occurs [3-7]. The mechanisms underlying these detrimental effects are unknown but may involve sex differences in inflammation that manifest with aging [8]. Modeling sex differences in the laboratory is difficult, as age and gonadal senescence contribute to outcomes [9]. The vast majority of pre-clinical stroke studies have used young animals with surgical gonadectomy to study sex differences. It is increasingly appreciated that tissue damage and functional outcomes after stroke are influenced by biologic sex in addition to the hormonal milieu [10-13]. The sex chromosome complement may contribute to sexual dimorphism; however earlier studies in young FCG mice found that hormones were the major contributor to stroke outcome [14]. Mice with ovaries that produced estrogen were protected, regardless of the sex chromosome compliment.

**Figure 1:**
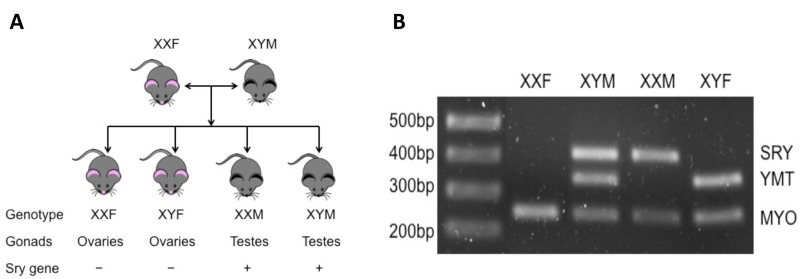
Brief introduction of FCG mice (**A**) Totally four genotypes of mice are produced by mating XXF to XYM mouse. XXF/XYF mice are gonadally females and XXM/XYM are males; among them, only XXF and XYM are fertile. (**B**) PCR results of FCG mice. MYO, autosomal gene (myosin) to confirm that PCR works (~250bp); YMT, Y gene to confirm presence of Y chromosome (~350bp); SRY, sex-determining region Y gene (~420bp).

Aging is the most important independent risk factor for stroke [15], and the response to stroke differs in aged animals. The acute activational effects of hormones on stroke in aged populations are minimal as circulating hormone levels are low. However, the organizational effects of hormones patterned throughout life add complexity to the sexual dimorphism seen in stroke. To specifically dissociate the activational/organizational effects of hormones from sex chromosome effects, we used the “four core genotype” (FCG) mouse model in our studies (Fig. 1). FCG mice are produced by mating C57/BL6 WT female mice (XXF) and XY^−^*Sry* males (XYM) in which the testes determining gene (Sry) is deleted from the Y chromosome and inserted onto an autosome. Four lines of mice are therefore generated including XX gonadal males (XXM; which have the Sry on an autosome and thus develop testes) or females (XXF; wild type), XY gonadal males (XYM; with the Sry on an autosome instead of the Y chromosome) or females (XYF; which have the Y chromosome without Sry and therefore develop as phenotypic females) [16].

Post-stroke inflammation leads to secondary neuronal damage following the initial ischemic injury. In prior work, we found a sex difference in the inflammatory responses even with equivalent ischemic injury between males vs. females after neonatal hypoxic-ischemic injury [17]. In aged wild-type cohorts, female mice also showed increased serum levels of monocyte chemotactic protein (MCP-1), IL-6, and TNF-α than males after stroke [9]. Since hormone levels are low at both ends of the age spectrum, we hypothesize that genetic sex difference exists in post-stroke inflammation. In the present study, we examined the contribution of chromosome complement to stroke outcomes and the immune response.

## RESULTS

### Stroke outcomes in FCG mice

**Figure 2:**
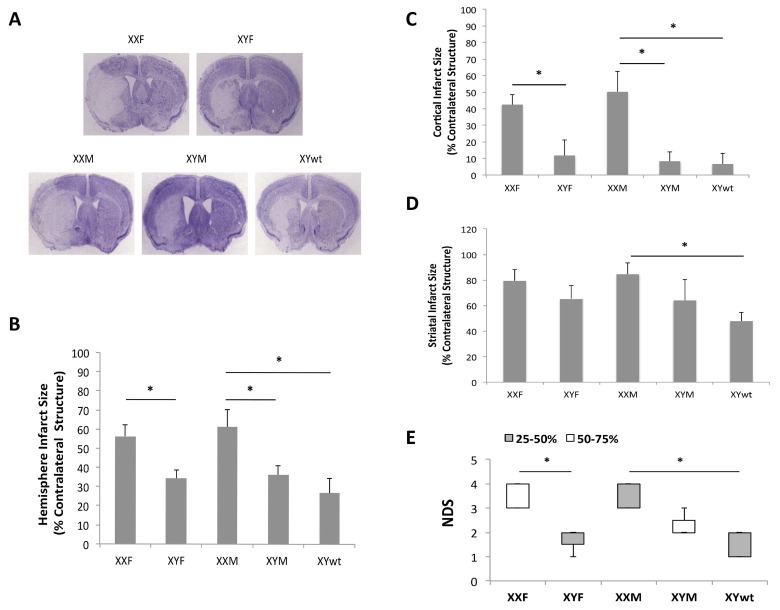
Stroke outcomes of 72 hours after MCAO (**A**) Representative CV staining pictures of stroked brain slices from FCG plus wide type male mice. (**B**-**D**) Quantitative data of infarct size in total hemisphere (B), cortex (**C**), and striatum (**D**). (**E**) Box-whisker figure of NDS. NDS was indicated as Median (interquartile range); white and gray boxes indicate the 25-50% and 50-75% interquartile range respectively; whiskers indicate the maximum and minimum NDS score. n=7~9 animals/group; *P < 0.05.

We first examined infarct volumes and neurological deficits in all four strains of FCG mice plus WT males 72 hours after MCAO. XXF mice had significantly larger infarct volumes in both the total ipsilateral hemisphere and cortex compared to XYF mice (n=7 animals/group; *P* < 0.05) (Fig 2A-C) suggesting a potential detrimental effect of the second X chromosome. No significant difference was found between XXF and XXM mice implying no effect of the gonads (ovaries vs testis respectively). Infarcts were also significantly larger in XXM mice than that in XYM or XYwt mice (n=7~9 animals/group; *P* < 0.05) (Fig 2A-C), consistent with a potential detrimental effect of the second X chromosome. The significant differences in infarction were found primarily in the cortical area but not in the striatum (Fig 2D). An equivalent infarction was seen in XYM vs. XYwt mice. Significantly higher NDS scores were seen in XXF vs. XYF and in XXM vs. XYwt mice (Fig. 2E). There was a trend towards a NDS increase in XXM vs. XYM group, but the difference did not reach the statistical significance. No differences in pH, pO_2_, pCO_2_, blood glucose, mean arterial blood pressure (MABP), or cerebral blood flow (CBF) by laser Doppler flowmetry were seen between any of the cohorts (Table 1).

**Table 1:** Physiological measurements in FCG mice

Group	pH	pO_2_ *mmHg*	pCO_2_ *mmHg*	Glucose *mg/dl*	MABP *mmHg*	LDF(%) *ischemia*	LDF(%) reperfusion
XXF	7.27 ± 0.15	90 ± 10	38 ± 12	163 ± 28	57 ± 7	11 ± 2	90 ± 5
XYF	7.29 ± 0.14	94 ± 12	41 ± 11	160 ± 30	68 ± 9	12 ± 3	89 ± 2
XXM	7.35 ± 0.06	87 ± 16	42 ± 11	168 ± 11	66 ± 7	13 ± 2	87 ± 6
XYM	7.37 ± 0.18	98 ± 15	41 ± 7	158 ± 20	63 ± 10	10 ± 2	89 ± 5
XYwt	7.31 ± 0.18	93 ± 12	38 ± 8	152 ± 25	58 ± 7	10 ± 2	91 ± 4

No differences were seen in physiologic variables and LDF between any two strains of FCG mice prior to (not shown) or 60 minutes after MCAO.

### Serum hormone and cytokine levels after stroke

**Figure 3:**
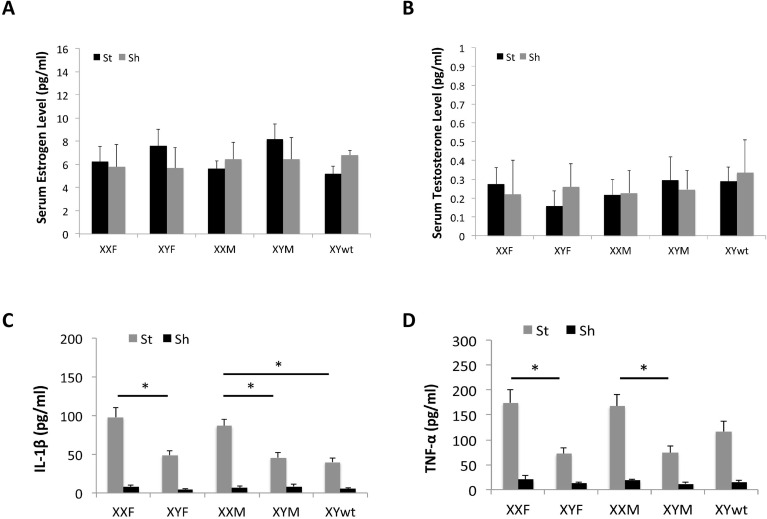
Serum levels of hormones and cytokines by Elisa Estrodial (**A**) and testosterone levels (**B**) showed no differences between any two strains either in sham or stroke mice. (**C**) IL-1β level. (**D**) TNF-α level. n=6/sroke group; n=4/sham group; *P < 0.05.

To investigate whether there were differences in gonadal hormone levels or in serum inflammatory markers that could contribute to the differential ischemic injury among the FCG mice, we measured serum estradiol, testosterone and cytokine (IL-1β and TNF-α) levels at terminal endpoints. Two-way ANOVA results showed that no significant differences in either estradiol (E2) (Fig. 3A) or testosterone (TT) (Fig. 3B) levels in the FCG cohorts or between stroke and sham groups (n=6/stroke group and n=4/sham group; *P* > 0.05). However, the cytokine levels exhibited a significantly different pattern. In each strain, the stroke group had significantly higher cytokine levels (either IL-1β or TNF-α) than sham groups (Fig. 3C&D). In stroke mice, levels of IL-1β level were significantly higher in the XXF vs. XYF group and this was also seen in the XXM group, which had significantly higher IL-1β levels than either XYM or XYwt group (Fig. 3C). A similar pattern was seen in TNF-α levels, with significantly higher levels in XXF vs. XYF and in XXM vs. XYM mice (Fig. 3D). The TNF-α level also trended higher in the XXM vs. XYwt group, although this difference was not significant. Taken together, the profile of circulating cytokine levels matches that of stroke outcomes.

### Microglial responses to stroke

**Figure 4:**
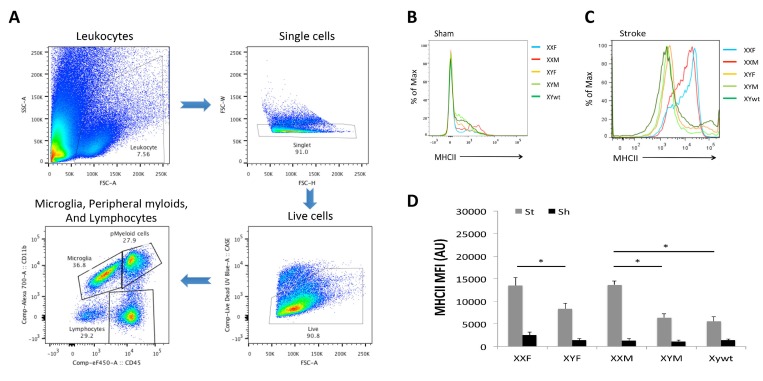
Microglial responses after stroke (**A**) Gating strategy indicating microglia as CD45^low^CD11b^+^, peripheral myeloid cells as CD45^high^CD11b^+^, and lymphocytes as CD45^high^CD11b^−^. (**B**) and (**C**) Representative histograms showing MHC II expression on microglia in stroke and sham groups respectively. (**D**) Mean fluorescence intensity (MFI) of MHC II in each groups. n=6/sroke group; n=3/sham group; *P < 0.05.

In order to determine if the differential cytokine profile seen in mice with a second X chromosome was secondary to sex differences in innate immunity in either the brain (microglia) or the periphery (monocytes and neutrophils) we examined infiltrating leukocytes in the ischemic brain by flow cytometry 3 days after stroke. The gating strategy is shown in Figure 4A. First we quantified microglia by gating on the CD45^low^CD11b^+^ population [18]. We found no differences in the absolute number of microglia between any of the groups. Next we examined the expression of microglial MHC II that functions to promote innate immune responses and is frequently used as a pro-inflam-matory marker [19-22]. Mean fluorescence intensity (MFI) of MHC II was quantified to determine the activation status of microglia (Fig. 4B-D) as MFI of MHC II has been frequently measured as an indicator of phagocyte activation [23, 24]. All five strains had equivalent baseline MHC II expression in sham mice (Fig. 4B); however, MHC II was differentially expressed after stroke. XXF mice had significantly higher MHC II MFI than XYF mice, and the MFI in XXM group was significantly higher than that in either XYM or XYwt group (Fig. 4C&D), the same pattern seen in infarct volume.

### Infiltrating peripheral immune cells in the ischemic brain

**Figure 5:**
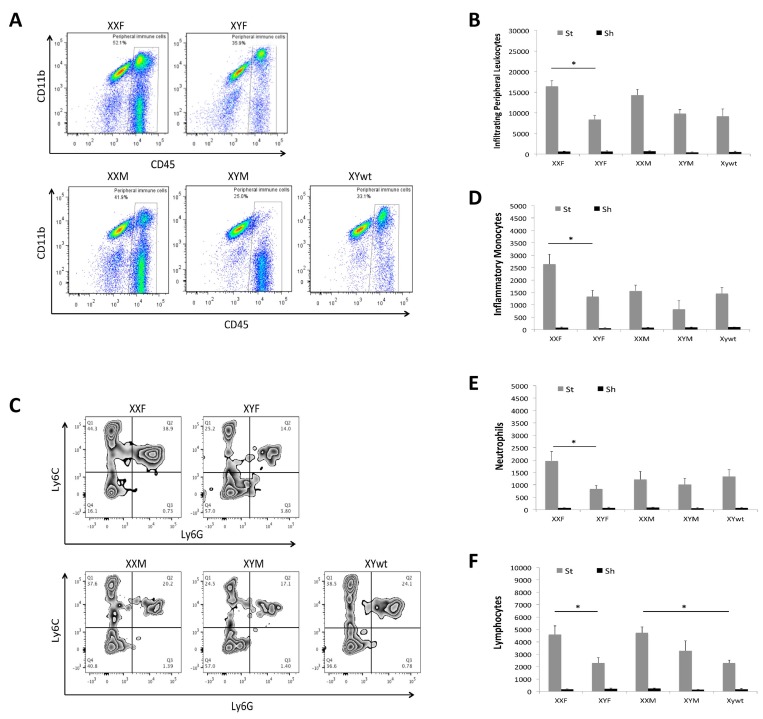
Infiltrating leukocytes in ischemic brains (**A**) Representative flow plots from each mouse strain showing gate strategy for infiltrating peripheral leukocytes (CD45^high^). (**B**) Total numbers of infiltrating peripheral leukocytes. (**C**) Representative plots indicating the percentage of inflammatory monocytes (Quardrant Q1) and neutrophils (Quardrant Q2) in infiltrating peripheral myeloid cells. Total numbers of each cell population were quantified in (**D**) monocytes, (**E**) neutrophils, and (**F**) lymphocytes. n=6/sroke group; n=3/sham group; *P < 0.05.

We also examined the composition of infiltrating leukocytes in the brain after stroke with flow cytometry 3 day after stroke. Peripheral immune cells (PIC) were defined as a CD45^high^ population that includes monocytes, neutrophils and lymphocytes and the absolute number of PICs in the ischemic brain was quantified (Fig. 5A&B). XXF mice had significantly more PICs than XYF mice, and a similar pattern was seen between XXM and XYM/XYwt mice although the difference was not significant. Next each component of PICs was evaluated. As shown in Figure 4A, we first gated on peripheral myeloid cells (CD45^high^CD11b^+^), which were further gated to inflammatory monocytes (Ly6C^+^Ly6G^−^; Quadrant Q1 in Fig. 5C) and neutrophils (Ly6C^+^Ly6G^+^; Q2 in Fig. 5C). These two populations in XXF mice significantly outnumbered that in XYF mice. No significant difference was seen in XXM vs. XYM or XTwt groups (Fig. 5D&E). Lymphocytes were gated as in Figure 4A. The quantification data showed significantly more lymphocytes in XXF vs. XYF and in XXM vs. XYwt mice (Fig. 5A&F).

## DISCUSSION

This study, which examined the effect of ischemic stroke sensitivity in aged FCG mice, reveals several important and novel findings on the contribution of sex chromosomes to stroke sensitivity. First and foremost, chromosome compliment is the primary contributor to stroke sensitivity in aged mice. Equivalently low serum levels of hormones (E2 or TT) were seen among all strains of FCG aged mice, but significant differences in stroke outcomes were found between gonadal female (XXF vs. XYF) or male mice (XXM vs. XYM/XYwt).

Mice that had a second X chromosome (XXF and XXM) had larger strokes independent of the gonadal phenotype (ovaries and testes respectively). The immune system also responded to ischemic injury in a chromosome-specific manner. Circulating levels of pro-inflammatory cytokines such as IL-1β and TNF-α were higher in XX mice and mirrored the pattern of infarct severity. Different components of the immune system showed differential characteristics in their responses to stroke. Microglia were significantly more activated in XXF vs. XYF and in XXM vs. XYM/XYwt mice, suggesting that chromosomes, rather than hormones, are the primary driver of ischemic outcome in senescent mice. Infiltrating peripheral leukocytes exhibited a similar pattern, with the most striking difference between gonadal females (XXF vs. XYF).

Sex differences in stroke are evident in both clinical and experimental studies, which are largely attributed to estrogen's neuroprotective effects [25]. E2's counterpart, TT, also has a significant impact on stroke, although this is more controversial as both beneficial and detrimental effects have been reported [26]. Clinically, in elderly populations that have low levels of gonadal hormones, women have higher stroke incidence and worse outcomes than age-matched men, suggesting that gonadal hormones are not the sole determinant of stroke outcome. Our previous studies in aged animals also found that aged females have larger infarcts than aged males after middle cerebral artery occlusion, despite equivalent levels of E2 or TT [9]. Gonadal hormones have both organizational and activational effects; the former is permanent and can occur in utero, whereas the latter is dependent on the acute circulating hormone levels [27, 28]. As the animals in the present study are aged and have equivalent circulating hormone levels, it is unlikely that activational effects exist that contribute to the stroke sensitivity. Organizational effects also seem to be minimal as no difference in stroke outcome was seen in XXF vs. XXM or in XYF vs. XYM/XYwt strains even though XXF/XXM (or XYF/XYM/XYwt) mice have differently established gonadal hormones at the embryonic level. The present study in aged FCG mice is in contrast to our previous work on young FCG mice [14], where sex differences in ischemic sensitivity appear to be shaped by organizational and activational effects of sex hormones rather than sex chromosomal complement. The controversy of findings between young and aged FCG mice suggests that the effects of hormones are critical in young brains' response to stroke however, become less important over time when brains age.

Two main chromosomal factors are involved in the FCG mice: the chromosomes ( X vs.Y), and the testes determining gene Sry. Since XXF/XXM (or XYF/XYM/XYwt) mice have the same stroke phenotype regardless of the presence of Sry gene only in XXM and XYM/XYwt genomes, a conclusion can be reasonably made that Sry contributes little to stroke sensitivity in aged brains. It has been reported that the X-chromosome has a different pattern of gene expression in women compared with men after ischemic stroke [29]. Our data showing different stroke outcomes in XXF vs. XYF and XXM vs. XYM/XYwt mice provide strong experimental evidence that similar to clinical studies, the X chromosome dosage is a key element in ischemic sensitivity that manifests in the aged. Interestingly, one of our previous studies has found that infarct sizes were equivalent between XX and XO female mice [30], indicating that the influence of X chromosome dosage on ischemic sensitivity is small. However, that study was done in young mice that bear overwhelming impact of organizational/activational hormone effects that chronically subside in aged brains. One possible mechanism may explain why the second X chromosome contributes to stroke sensitivity in aged brains: Gene escape from X-chromosome inactivation (XCI) [31]. Normally males and females differ in sex chromosome content (XY vs. XX) and X chromosome imbalance is tolerated because of dosage compensation by XCI [32, 33]. With aging, XCI becomes unstable and some genes that were subjected to XCI may escape X inactivation [34, 35]. The stroke sensitivity seen in XXF/XXM mice could possibly be caused by the escaping genes that are crucial in brains' response to stroke. This theory has been frequently proposed to explain for the phenotype spectrum of disease in females and suggests a role in sex differences seen in human diseases [36]. The study on the effect of gene escape from XCI on stroke has opened up a brand new field in this lab and genomic analysis in the aged ischemic brains of both sexes will be performed. It is less likely that Y chromosome genes contribute to the better outcomes in XYF/XYM/XYwt compared with XXF/XXM mice as the phenomenon of “male-sensitivity” in stroke has been well documented in young adults [26]. The present study strongly suggested that chromosomal effects contribute to the stroke sensitivity in aged animals; however, it is not conclusive yet at this moment whether and how X chromosome genes influence stroke outcomes. Future work in this field is warranted.

Innate immune responses play a critical role in the pathophysiology of ischemic stroke [37]. Microglial activation after stroke has been increasingly recognized as a key element in initiating and perpetuating the immune response. Microglia are the predominant cell population in the infarct area compared to monocytes, and are the first immune cells activated after brain injury [38]. The present data showed the microglial response represented by MHCII expression has the same pattern as that of stroke outcomes, indicating microglial activation also took on a chromosomal effect. Interestingly the serum levels of IL-1β and TNF-α exhibited the same pattern as that of MHC II on microglia, and the infiltration of peripheral leukocytes also showed the similar pattern, especially the pattern of lymphocytes that exhibited a close correlation to the stroke outcomes (Fig. 5F). The contribution of specific lymphocyte cell type to stroke sensitivity will be further explored in our future studies. The inflammatory response is both resultant and causative to the ischemic injury, and exerts the secondary neuronal damage [17, 39, 40]. The chromosomal effect seen in the inflammatory responses may be secondary to the infarct; however, our previous study with hypoxic-ischemia model found equivalent infarct lesion in male and female animals led to a sex-specific pattern of inflammatory responses [17]. Base-line sex differences were found in the expression of microglial markers and membrane receptors that are critical for regulating microglial functions [41, 42]. Immune responses are shaped by biological differences between males and females, not only by hormonal influences but also genetic effects [43]. Approximately 1000 genes are located on the X chromosome, many of which are responsible for the immune function [44]. Whether these X-linked genes mediate different levels of immune responses between males and females needs to be explored. The relocation of Sry gene from Y chromosome to autosome 3 may also influence the infiltration of peripheral myeloid cells into the ischemic brain, as our data showed decreased CD45^high^/CD11b^+^ cell populations in XYM and XXM mice although the total infiltrating peripheral leukocytes still demonstrated the effect of the second X-chromosome (Fig 5). The interaction between Sry and immunity will be pursued in future studies. It can be concluded from our data that XY aged mice have attenuated inflammatory responses compared to the XX counterparts, and this is consistent with our [45] and others' [46] previous studies that showed WT aging male mice brains have strongly diminished pro-inflammatory responses compared to females after stroke. It is very likely that post-stroke immune responses are subject to sex-specific mechanisms in addition to ischemic insults.

In summary, aged brains respond to ischemia in a sex-specific way, and the chromosomal complement contributes to the sex difference. The chromosomal effect not only affects stroke outcomes, but also shapes the post-stroke immune responses. Although Sex differences in young stroke brains are dependent on the effects of gonadal hormones, the sexual dimorphism in aged stroke brains are more likely determined by the chromosomal complement.

## METHODS

### Experimental animals

C57BL/6J XYM mice were provided by Dr. Arthur Arnold from UCLA. XYM mice were bred to C57BL/6J wild-type females (XXF) purchased from Jackson Laboratory. A cohort of Wild-type C57BL/6J male mice (XYwt) (Charles River Laboratories; NIA, Aged Mouse Colony) was included in the study as a control for the transgenic (on chromosome 3) vs. endogenous Sry (on the Y chromosome). All mice were 18-20 months old. All experiments were performed according to NIH guidelines for the care and use of animals in research using ARRIVE guidelines and under protocols approved by the Animal Welfare Committee of UT Health and by the UCHC Animal Care and Use Committee.

### MCAO model and stroke outcome measurement

Focal transient cerebral ischemia was induced by 90-minute middle cerebral artery occlusion (MCAO) under isoflurane anesthesia as described previously [11] except that a 0.23 mm silicone coated suture was utilized to achieve occlusion [45]. Rectal temperature was maintained at approximately 37°C during surgery with an automated temperature control feedback system. In separate non-survival cohorts of all four strains, femoral arterial blood pressure and physiological measurements, including blood pH, pO2, pCO2, and blood glucose, were obtained. Cortical perfusion using Laser Doppler Flowmetry was evaluated throughout MCAO and early reperfusion as described previously [47]. Three days after MCAO, mice were euthanized and brain slices stained with cresyl violet (CV) staining as previously described [9]. Infarct volumes were quantified with Swanson's method [48] to correct for edema. The total volumes of both contralateral and ipsilateral hemisphere, and the volumes of the striatum, cortex in both hemispheres were measured and the infarct percentage was calculated as % contralateral structure to avoid mis-measurement secondary to edema. Neurological deficit scores (NDS) were recorded at sacrifice. The scoring system was as follows: 0, no deficit; 1, forelimb weakness and torso turning to the ipsilateral side when held by tail; 2, circling to affected side; 3, unable to bear weight on affected side; and 4, no spontaneous locomotor activity or barrel rolling.

### Flow cytometry

Leukocytes from brain tissue were prepared as previously described [8, 17, 38]. Animals were anaesthetized with Avertin (2, 2, 2-Tribromo-ethanol) and intracardially perfused with phosphate-buffered saline (PBS) for 5 minutes. Brains were harvested and dissected to isolate the ipsilateral stroke/sham hemisphere. The whole ipsilateral hemisphere was placed in RPMI 1640 complete medium (10% fetal calf serum, 1% sodium pyruvate, 1% non-essential amino acid, 0.1% β Mercaptoethanol, 100 U/ml of penicillin and 100 μg/ml of streptomycin) in separate tubes on ice. The brains were mechanically dissociated and incubated with 100 μl of collagenase/dispase (1 mg/ml, Roche Diagnostics) and 300 μl DNAse I (10 mg/ml, Roche) for 45 min at 37°C. After incubation, the brain homogenate was passed through a 1 ml pipette tip several times and harvested in 20 ml complete RPMI. The cells were pelleted at 1200 G, 4°C for 10 min, re-suspended in 40 ml complete RPMI, passed over a 70 μm cell strainer and pelleted again. The filtered cells were re-suspended in a 70%/30% Percoll gradient (GE Healthcare) and spun at 2000 rpm for 25 min at room temperature with no brake. Myelin was removed and cells collected from the interface into 12 ml complete RPMI. The cells were washed and re-suspended in 300ml FACS buffer for antibody staining and counting. All samples were run on low flow rate until zero events were reached to obtain the absolute numbers of leukocytes in the hemisphere. Fluorophore-conjugated antibodies against CD45 (#8017-9459), CD11b (#56-0112), Ly6G (#48-5931), Ly6C (# 45-5932), and MHCII (#11-5321) were obtained from eBioscience.

### ELISA

Blood was extracted and centrifuged at 6,000 rpm for 10 minutes at 4°C. The supernatant was collected to isolate serum from the blood. Hormone levels were measured using ELISA kits for testosterone (#TE187S-100; Calbiotech) and estrogen (#BQ180S; BQ Kits). The serum concentration of TNF-α and IL-1β was determined by mouse TNF-α (#88-7324) and IL-1β (#88-7013) ELISA kits (eBioscience).

### Statistics

Data from individual experiments are presented as Mean ± SEM and analyzed with one-way (infarct volumes) or two-way ANOVA (hormone levels, cytokines, and flow cytometry data). The ordinal data of NDS are analyzed with Mann-Whitney U test. P< 0.05 was considered statistically significant. Investigators were blinded to mouse strains for stroke surgery, NDS scoring, infarct, flow cytometry and inflammation analysis.
